# Attitudes of Nursing Home Staff towards Influenza Vaccination: Opinions and Factors Influencing Hesitancy

**DOI:** 10.3390/ijerph17061851

**Published:** 2020-03-12

**Authors:** Francesca Moretti, Donatella Visentin, Elena Bovolenta, Michela Rimondini, Silvia Majori, Mariangela Mazzi, Albino Poli, Stefano Tardivo, Emanuele Torri

**Affiliations:** 1 Department of Diagnostics and Public Health, Section of Hygiene and Preventive Medicine, University of Verona, 37134 Verona, Italy; donatellavisentin77@gmail.com (D.V.); elena.bovolenta@gmail.com (E.B.); silvia.majori@univr.it (S.M.); albino.poli@univr.it (A.P.); stefano.tardivo@univr.it (S.T.); 2 Department of Neuroscience, Biomedicine and Movement Sciences, Section of Clinical Psychology, University of Verona, 37134 Verona, Italy; michela.rimondini@univr.it (M.R.); mariangela.mazzi@univr.it (M.M.); 3 Department of Health and Social Policy, Autonomous Province of Trento, 38121 Trent, Italy; emanuele.torri@provincia.tn.it

**Keywords:** flu vaccination, staff hesitancy, nursing home, immunization promotion

## Abstract

Seasonal influenza is recognized to be a significant public health problem and a cause of death, especially in fragile persons. In nursing homes (NHs), vaccination for both residents and staff is the best preventive strategy. However, professionals’ immunization rates are far from reaching the international recommended values. This study aims to describe the adherence and attitudes of NH staff towards flu vaccination and to explore staff hesitancy. A questionnaire was developed based on a literature review and on the 3Cs (confidence, complacency, convenience) of the WHO framework and administered among the staff of four NHs of a province in the northeast of Italy. Results demonstrated a low adherence towards annual vaccination (i.e., only 3% declared getting the flu vaccination each year). Complacency, confidence and convenience all showed a significant impact on the attitude towards vaccination both in univariate and multivariable analysis, with complacency being the most strongly associated area. The area of confidence resulted in strongly challenging factors. Only 24.8% of interviewees appeared trustful towards the efficacy of receiving immunization and 34% declared safety issues. Insights from the study can support the implementation of effective interventions to improve vaccination adherence in NHs. Specifically, increasing complacency by raising awareness related to the risks of influenza appears to be an essential strategy to effectively promote vaccination uptake.

## 1. Introduction

Seasonal influenza is recognized as a significant public health problem and an important cause of death and hospitalization, especially in fragile persons [1]. In nursing homes (NHs), micro-epidemics can spread and lead to potentially serious consequences for residents and workers [2]. In this setting, influenza vaccination for both residents and staff is the best preventive strategy to reduce the epidemiological, clinical, and economic impact of influenza. Although residents’ coverage rates are usually high (>85%), this is not sufficient to ensure protection since the elderly respond poorly to vaccination due to immunosenescence [3,4]. Thus, influenza vaccination among NH employees may become an important indirect protection strategy [5].

Nevertheless, professionals’ immunization rates are far from reaching the values recommended by the Italian Vaccine Preventive Plan 2017–2019 (a minimum of 75% and optimal 95% among risk groups including people over 65 and healthcare workers) [6]. Among EU Member States, the median influenza vaccination coverage rate among healthcare workers (HCWs) in 2016–17 was 30.2%, with percentages between 25%–55.9% in the long-term care facility (LTCF) setting [7,8,9,10]. For Italy, studies have reported even lower vaccination coverages (ranging from 14% to 25%) [8,9,10], even if the flu vaccine is offered free of charge by the National Health Service (NHS) to all groups at risk including HCWs.

Reasons for such a low influenza vaccination uptake may include a wide range of factors that can be encompassed within the phenomenon of vaccine hesitancy. According to the Strategic Advisory Group of Experts Working Group on Vaccine Hesitancy (SAGE-WG), the concept refers to “delay in acceptance or refusal of vaccination despite availability of vaccination services” [11]. The SAGE-WG underlines that defining hesitancy is “complex” and that, between the two opposing positions of a full acceptance versus a firm refusal of all recommended immunizations, there is a wide range of attitudes that brings people to accept/refuse only some vaccinations or accept/refuse vaccination without being sure of their decision, or the attitude may lead people to delay vaccination. All these behaviours reflect a different level of hesitancy that may be influenced by several factors. In order to facilitate the understanding of this hesitancy, the SAGE-WG developed a framework based on the “3Cs” model (complacency, convenience, confidence) of the World Health Organization (WHO) [11]. Specifically, according to this model, hesitancy is influenced by factors, such as the low risk perceptions of vaccine-preventable disease (complacency), the presence of obstacles to convenient access to vaccination, and poor confidence in vaccines (e.g., fear of adverse events, concerns about efficacy). Moreover, the SAGE-WG developed a determinants matrix that further recognizes factors affecting hesitancy, differentiating between contextual influences (e.g., socio-cultural, environmental, health system/institutional, political factors), individual-group influences (e.g., personal perception and social environment) and vaccine/vaccination-specific issues (e.g., factors directly related to vaccination such as vaccination schedule, mode of administration). Contextual influences underline the potential role of settings and local characteristics in affecting hesitancy [12]. However, to our knowledge, no published data are currently available on hesitancy determinants among NHs in the Italian context.

The aims of the present study are twofold:

- To describe adherence and attitudes of NH staff towards flu vaccination;
- To explore staff hesitancy and its relationship with the attitude towards flu vaccination.

Insights arising from our study may support the implementation of effective interventions to increase vaccination adherence in NHs.

## 2. Materials and Methods 

### 2.1. Instrument: NH Staff Survey

A questionnaire to explore staff hesitancy towards the influenza vaccine was developed based on a literature review and on the 3Cs WHO framework. Specifically, a list of potentially relevant items representing factors influencing hesitancy was selected and classified according to the 3C model. The survey included 12 items evaluated on a five-point Likert rating the level of agreement (from strong disagreement to strong agreement) with a set of statements regarding confidence, complacency and convenience regarding flu vaccination. An additional item (rated on the same five-point Likert scale) was developed to explore staff attitude towards flu vaccination (Item 13—NH staff should have flu vaccinations each year). Moreover, two questions explored the adherence towards influenza vaccination practice (Q1. “How many times did you get vaccinated for influenza in the last 3 years?”) and the presence of advice from a physician (Q2. “Have you ever been advised by a physician to get a flu vaccination?”). Finally, respondents were asked about their professional roles (Q3) (Appendix A).

### 2.2. Population and Data Collection

This project is part of a wider study aiming to explore the safety culture among staff in NHs. This study was conducted between June and July 2018 in four public NHs of the Autonomous Province of Trento, a region in north-eastern Italy. The population included not only HCW but also all other professionals working in the facility (e.g., support staff, administrative staff) for a total of 437 persons. All directors of the NHs were extensively informed regarding the main aims of the project and asked to fill out an agreement form to conduct the study in the respective facility. The most effective mode of informing the staff and encouraging participation was established within each NH (e.g., emails addressed to all the personnel, staff meetings, brochures). Specifically, a few days before the beginning of the survey, people were informed (through one of the selected channels) of the main purposes of the study, and indications were given regarding how to complete and return the questionnaire. To further promote the survey, a reminder with relevant information about the project, including researchers’ contact details for potential questions, was displayed on notice boards in each NH. The survey was paper-based and anonymous. In each NH, a head nurse was identified as a referent of the project and committed to distribute the survey to the staff (e.g., during daily meetings). A locked drop-box was available to facilitate the collection of returned surveys. A data collection duration of maximum 4 weeks was established. Every week, one of the researchers went to each NH to collect the returned questionnaires and to calculate a preliminary response rate (RR). A soliciting e-mail was sent by the director of the NH after the first and second week to maximize the RR. 

### 2.3. Data Assessment and Statistical Analysis

According to the “3C” model, three latent factors were identified by calculating the average score of the following groups of items: Item 1, Item 2, Item 7, and Item 8 for confidence, Item 3–Item 6 for Complacency and Item 9–Item 12 for convenience. 

A confirmatory factor analysis was performed to assess the validity of the model based on the three latent constructs. Scores for Item 7, Item 8, and Item 10 were reversed according to their formulation. The Confirmatory Factor Analysis, following the approach of the Structural Equation Model with Satorra–Bentler (SB) adjustments in order to take into account some non-normally distributed items, showed that the indices of confidence, complacency and convenience were well supported: root mean square error of approximation (RMSEA-SB) = 0.051, standardized root mean square residual (SRMR) = 0.037, coefficient of determination (CD) = 0.958, Comparative Fit Index (CFI-SB) = 0.997, Tucker–Lewis index (TLI-SB) = 0.991. 

The reliability properties for each latent factor, investigated using Cronbach’s alpha, were 0.76 (range: 0.61–0.86, excluding one item at a time) for confidence, 0.72 (0.51–0.79) for complacency and 0.29 (0.12–0.35) for convenience. The sample was then divided into two groups according to the frequency of flu vaccination practice (question Q1): (i) people performing vaccinations each year in the last three years, representing the group of “fully convinced” about flu vaccination; (ii) people performing vaccinations less than three times in the last three years, representing the vaccine hesitant/refusing group.

Descriptive statistics were used to explore the distribution of traits among the vaccine-hesitant subsample. Determinants of hesitancy were analysed by exploring the relationship between the score distribution of a set of variables (independent variables) on attitude towards vaccination (i.e., the score distribution of Item 13 as dependent variable). A set of preliminary analyses was performed to assess both the distribution of Item 13 and the potential influence of the nested structure of the sample (responders are nested in NHs) on the target information: the skewness–kurtosis test was used to confirm the normal distribution (Chi^2^(2) = 2.5; *p* = 0.29), while ANOVA and the intraclass correlation coefficient (ICC—through a multilevel model approach) were used to verify the homogeneity of the mean scores among the four NHs. A set of linear regression models was then performed to identify which one best explained the relationship between attitudes towards vaccination and the identified independent variables.

The level of significance was set at 0.05. Statistical analyses were performed using STATA 15.

## 3. Results

### 3.1. Survey Response Rate

**Figure 1:**
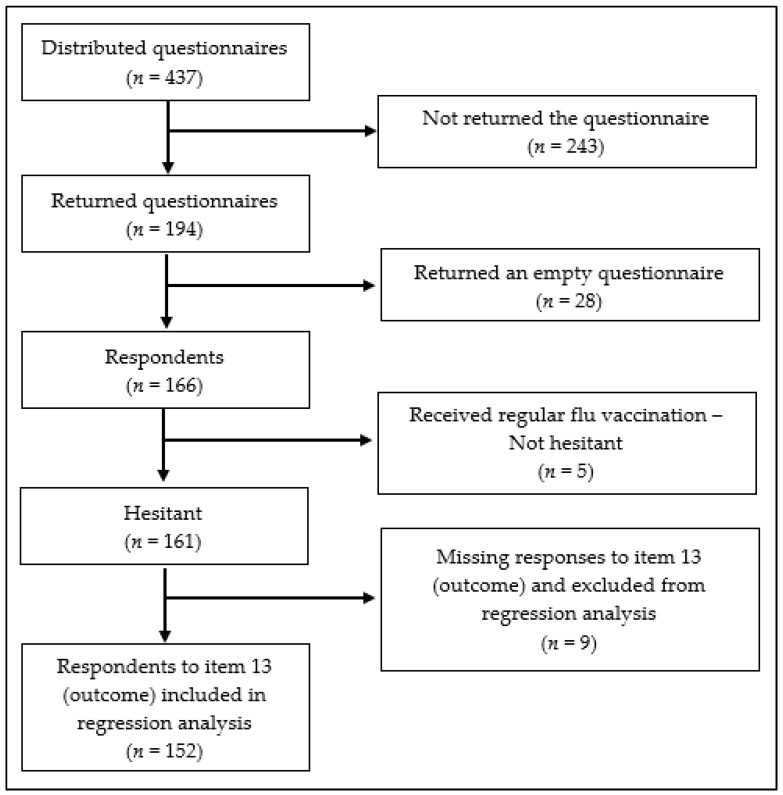
Flow chart of the entire recruitment process.

Of the 437 distributed questionnaires, 194 were returned (44.4%). However, 28 were returned empty (6.4%) and thus excluded from further analysis, leaving a total sample of 166 respondents (38.0% response rate, RR). Figure 1 illustrates the entire recruitment process. The RR varied among the nursing homes, ranging from 28.3% to 54.5%.

Table 1 shows the sample description and RR per staff position.

### 3.2. Adherence and Attitude towards Flu Vaccination

Only 5/166 (3.0%) declared having a flu vaccination each year, and 16/166 (9.6%) reported to have had at least one flu shot in the last three years. In total, 137/166 (82.5%) of the employees worked directly with residents (i.e., direct care staff, nurses and other healthcare providers). The proportion of participants vaccinated at least once in the last 3 years was significantly lower in this group compared to employees not working directly with residents (8.7% vs 26.3%, chi2(1) = 4.14; *p* < 0.05).

**Figure 2:**
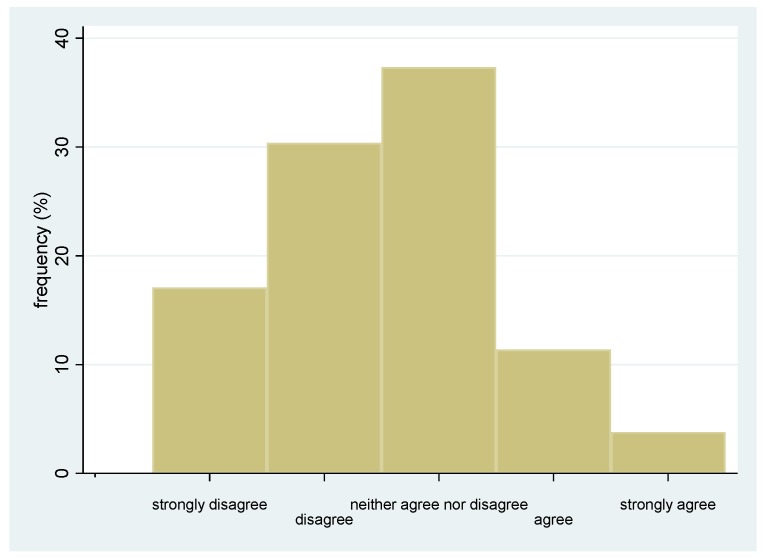
Frequency distribution of attitude towards vaccination (*n* = 152).

Excluding the five participants fully adherent to vaccination, the hesitant/refusing group included 161 subjects. Among these, 88/161 (54.7%) had not been advised by a physician to get the flu vaccination. Nine participants did not respond to the Item “Nursing home workers should get flu vaccination each year”, resulting in 152 respondents (Figure 1). Among these, 49% disagreed/strongly disagreed with the statement, 37% neither agreed nor disagreed, and only 14% agreed/strongly agreed (Figure 2).

### 3.3. Distributions of Factors Influencing Hesitancy towards Flu Vaccination

The frequency distributions of the 12 items representing factors influencing hesitancy towards flu vaccination varied widely between individuals vaccinated at least once in the last three years (*n* = 16) and individuals never vaccinated in the last three years (*n* = 145) (data not shown due to the low number of the first group which was not sufficient to get significant results; see Appendix B for descriptive statistics).

Table 2 shows the item distribution among the vaccine-hesitant sample (*n* = 161).

**Figure 3:**
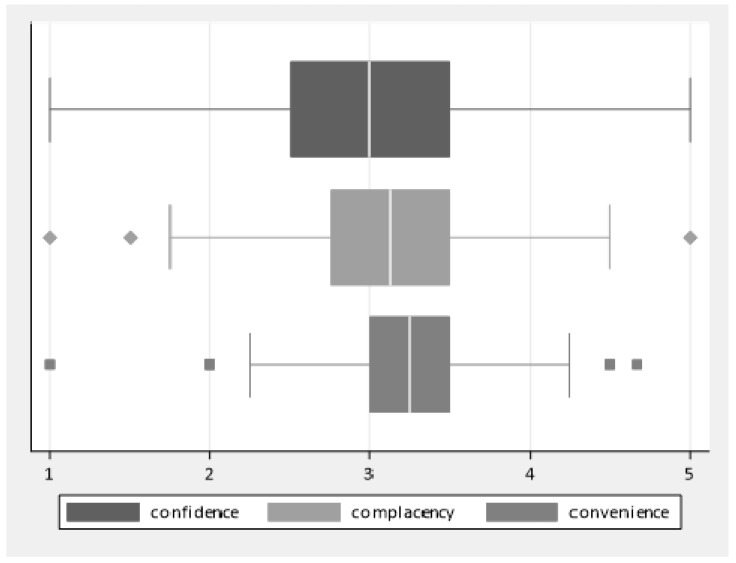
Box plots of the score distribution of the three factors.

Figure 3 shows score distribution of the three factors (Confidence, Complacency, Conveninece).

### 3.4. Relationship between Potential Determinants of Hesitancy and Attitude towards Vaccination

The explorations of scores related to the attitudes towards vaccination (Item 13) showed small differences among the mean values of each NH (range: 0.74–1.17); the ANOVA confirmed that they were not significant (F = 1.05; *p* = 0.37), and the intraclass correlation coefficient showed the homogeneity of the sample (ICC < 1), so we preferred to use a one-level approach to the whole sample, skipping the detail of cluster data.

A preliminary bivariate analysis was performed to explore the relationship between the impact on attitude towards vaccination (e.g., the score distribution of Item 13 “nursing home workers should get a flu vaccination each year”) of the following variables: being advised by a physician (question Q2), working directly with residents (identified and merged from question Q3), complacency, confidence, and convenience. Both Q2 and working directly with residents showed no significant relationship with attitudes towards vaccination (Table 3, column 3, *p* > 0.05), which means that they cannot influence the target variable (their relationship with attitudes towards vaccination was estimated at around zero: b = −0.16 and −0.38; see column 2). In the next step, a parsimonious final model was identified (Table 3; right part: multiple regression), exploring the partial effects of the relevant variables of complacency, confidence, and convenience (*p* < 0.05 in column 3). This final model could explain two-fifths of the variability of attitudes toward vaccination (adjR2 = 0.41), demonstrating that all the 3C components maintained their relationship with the attitude (Table 3, column 6, *p* < 0.05), albeit with a lesser intensity. It can be noted that a greater influence is assigned to complacency (b = 0.60, see column 5), followed by convenience (b = 0.38), and confidence (b = 0.25). The estimated coefficients also suggest potential efforts to increase the attitude toward vaccination. For instance, in order to modify the attitude from neutral to agreement (from 3 to 4 in the Likert scale), a healthcare professional could change both his/her level of compliance (+0.60) and of convenience (+0.38) by one unit as measured by the questionnaire.

## 4. Discussion

The survey aimed to explore the adherence, attitude and determinants of hesitancy towards flu vaccination of a sample of staff working in four different NHs of the Trento region. Even though the number of potential respondents was substantial, only a relatively small proportion of questionnaires was returned. This response rate seems to be related to a distrustful or uninterested attitude of the staff towards vaccination rather than to issues regarding inadequate advertising and the dissemination of the survey. Indeed, a great number of resources was dedicated to ensuring that each staff member was adequately informed and motivated to take part in the project. Moreover, during data collection, a small group of employees strongly advocated against vaccination. Indeed, these employees raised several issues concerning the survey to the leadership and did not perceive the survey as an instrument to explore staff attitude and opinions but as a first step to making the flu shot compulsory. Further, this questionnaire was distributed together with a safety culture questionnaire as part of a wider survey aiming to explore staff attitudes towards safety issues. It is worthy of notice that a relevant number of responders who did not fill the short questionnaire on vaccination fully returned the much longer part related to safety culture. Indeed, the RR for the safety culture questionnaire was much higher, with approximate values of 60% in the same NHs. The low RR recorded in this study seems to indicate that staff vaccination is a critical issue that needs to be carefully addressed, focusing specifically on the context of NHs. Similar studies exploring NH staff perceptions of flu vaccination throughout a survey found comparable response rates (e.g., from 10% to 45%), confirming the complexity of the topic [13,14,15,16]. This evidence is further underlined by the reported extremely low adherence to annual flu vaccination. A recent systematic review and meta-analyses on Italian HCW adherence to influenza vaccination found vaccination rates of approximately 13% among nurses and ancillary workers [17]. Moreover, in line with our results, a multicentre cross-sectional study conducted in 10 Italian cities reported a proportion of 4% [9]. The low percentage of vaccinated staff was observed in all four NHs. Indeed, multivariable regression showed that the attitude towards vaccination was not influenced by the facility. This result highlights the crucial role of creating an adequate culture of vaccination and increasing HCWs’ accountability towards such an important preventive measure in NHs. The US Center for Disease Control and Prevention (CDC) recommends a set of strategies to overcome the barriers of HCW towards the flu vaccination [18]. Specifically, the CDC underlines the need to “establish a culture of prevention within the organization” through several actions that can be implemented by staff managers, such as actively encouraging employees to get the vaccine (e.g., via e-mail, posters, newsletter, and any other communication tools) or “vaccinate the medical director and all managers in front of the staff”. Establishing such a culture of vaccination may help the staff to perceive the flu vaccine as a duty towards their patients, especially if they are frail, as in the setting of NHs. It is noteworthy that the staff who were not directly working with residents had a significantly higher (even if insufficient) proportion of being vaccinated. However, at multivariable analysis, working directly with residents did not influence the attitude towards vaccination.

Among the participants not regularly performing vaccination (representing the group of “hesitant” individuals according to the WHO definition), one-third had a neutral position (e.g., neither agree nor disagree) regarding the need to get a flu vaccination each year. This result is of particular importance for public health policy makers since it emphasizes that there is a wide opportunity to positively influence staff behaviour and increase vaccine coverage. On the other hand, approximately one-fifth of participants had a firm negative attitude towards this need. A study exploring both NH and hospital staff attitudes towards flu vaccination revealed that one of the main reasons for non-vaccination was the necessity of annual vaccination [13]. Such a regular uptake needs strong motivation, which is often impacted by doubts regarding the efficacy due to viral mutations and the difficulties in predicting the best composition of flu vaccine based on previous circulating strains, as highlighted in the literature including several systematic reviews [19,20,21,22]. Interestingly, one-fifth of respondents, despite agreeing with the importance to regularly get a flu vaccination, did not perform it, suggesting that the attitude itself is not sufficient to ensure an adequate vaccination coverage. Wide-reaching awareness campaigns should be launched with the careful selection of particularly important issues. 

Only half of our sample reported to have been advised by a physician to get the flu vaccination. In addition, this factor did not show a significant impact on the attitude towards vaccination in the multivariable analysis. This result indicates that individual vaccination promotion is still not sufficient and unable to reach the whole population at risk. As previously emphasized, according to the literature, a key role in promoting health behaviours is played by staff managers. General practitioners (GPs) may also have several opportunities to advertise to their patients, including HCWs, to regularly uptake flu immunization. However, prescribing habits are strongly influenced by a complex spectrum of psycho-social factors (Donisi et al., 2019). According to literature, some GPs show doubts regarding the necessity to get a flu vaccination, which can lead to difficulties in adequately promoting it [23,24]. 

Complacency, confidence and convenience all showed a significant impact on attitudes towards vaccination both in univariate and multivariable analysis. Out of these dimensions, complacency was the most strongly associated with attitudes. According to a recent systematic review on HCW barriers towards flu vaccination, issues related to complacency were frequently reported, even if a lack of confidence was shown to be the major obstacle [25].

In our study, the area of confidence showed the highest proportion of neutral scores and the lowest proportion of positive answers, showing that it may be the most challenging factor. Specifically, only one-quarter of respondents appeared trustful towards the efficacy of receiving immunization. These data might be related to the actual moderate effectiveness of flu vaccines due to antigenic drift and subsequent mismatch with circulation strains [26]. Indeed, in our sample, one-quarter of respondents agreed with the statement “I don’t think flu vaccination is useful” and approximately 40% neither agreed nor disagreed. Thus, to avoid data on effectiveness negatively affecting the perception of usefulness, it is essential to spread evidence-based information on the potential positive public health impact of the flu vaccine. For example, a recent metanalysis on the effect of trivalent flu vaccine estimated that over a period of 50 years, immunization would have avoided more than 37 million influenza episodes, 476,000 influenza-related hospitalizations, and 67,000 influenza-related deaths [27]. On the other hand, the proportion of respondents concerned about adverse events was equal to those not worried or neutral about this issue (one-third of each group). The well-known case of the trivalent inactivated flu vaccine FLUAD^®^ highlighted the negative impact and potential dramatic consequences of misinformation regarding the safety of flu vaccination. Indeed, in November 2014, during the 2014–15 flu campaign, the Italian Medicines Agency (AIFA) suspended, as a precautionary measure, the use of two batches of the influenza vaccine FLUAD^®^ after the occurrence of three suspected deaths (AIFA, 27th of November 2014) [28]. Investigations performed by authorities excluded an association, and the two batches were reintroduced in December 2014 [29]. After this episode, a decrease by 80% of the number of vaccinated people (25%–30% of the overall 2014–2015 national immunization campaign) was registered [30]. According to experts, this decrease in flu coverage might have been one of the determinants of the excess of mortality (9.1%) observed in Italy in 2015, as compared to 2014, mainly regarding people 65 years and older [31]. Therefore, efforts should be directed towards improving communication on vaccine safety issues in order to overcome distrust and raise public confidence.

The dimension of complacency showed the strongest influence on attitudes towards vaccination. More than half of respondents did not consider flu a potentially dangerous disease and tended to underestimate the risk of getting it in the NH setting. Similar findings were retrieved in several systematic reviews exploring determinants and barriers of flu vaccination uptake [19,20,22]. The observed underestimation of the disease burden of influenza may be related to the widespread false knowledge that the flu infection is just a bad cold [32]. Despite this misperception, a recent modelling study estimated that the annual flu infection is associated with up to 9 per 100,000 deaths worldwide and that the highest rate is ascribable to persons 75 years or older (up to 100 per 100,000) [33]. Moreover, respiratory infections show a high attack rate in the NH setting due to prolonged close contacts among residents, their caregivers, and staff [34,35]. According to O’Connor et al., outbreak occurrence and duration are associated to lower staff flu vaccination coverage [34]. Despite this evidence, in our sample, only one-third of respondents showed awareness regarding the importance of getting the flu vaccine in order to indirectly protect residents, while almost half maintained a neutral position. Accordingly, it has been demonstrated that the opportunity to get a vaccination is perceived by HCWs as an individual decision rather than a duty to protect their patients and families from consequences of the flu [13]. Such a position may be related to the already mentioned distrustful perception regarding the efficacy of flu vaccination. Actually, a recent Cochrane review was unable to provide strong evidence supporting the effectiveness of NH staff flu vaccination in preventing influenza among residents [36]. On the other hand, a systematic revision of randomized trials, cohort studies, and case-control studies proved a significant protective association for influenza-like illness and laboratory-confirmed influenza [37]. Considering the well-known negative impact of influenza on the elderly and the established safety of flu vaccines, the risk–benefit balance strongly supports staff vaccination, as stated by all major healthcare authorities [38,39]. Moreover, both CDC and WHO have identified HCWs and other staff members working in LTCFs such as NHs as a priority group for influenza immunization.

The dimension of convenience includes a group of items exploring the perceived easiness and advantages of getting the influenza vaccination. According to our results, less than half of the sample declared having received adequate information regarding the benefits of getting vaccinated from influenza. Such a result again raises concerns regarding the level and quality of flu vaccination counselling performed by key professionals, such as staff managers of the facilities and GPs. On the other hand, easily accessible vaccination was considered as critical only by a small part of the sample (approximately 5%), confirming the importance of adequately informing and motivating the staff instead of solely giving the opportunity to get the vaccination.

Only one-tenth of respondents agreed on making the flu vaccination mandatory, while more than half of the sample disagreed with this statement. These contrasting positions towards compulsion were also observed in similar studies [13,40].

The evidence shows that a mandatory flu immunization program for HCWs may increase the vaccination rate, reaching an extremely high coverage of up to 98% [41,42,43]. However, compulsory vaccination may also have negative consequences. According to an experimental study exploring the effects of introducing partial compulsory immunization, being obliged to get vaccination increased the level of anger among individuals with a former negative attitude towards this prevention tool [44]. As a consequence, the wish to recover the denied right of choice led also to a refusal of voluntary vaccination. Furthermore, a comparable high level of vaccination coverage can be obtained also with well-organized voluntary immunization programs focused on increasing HCWs’ commitment [45].

### Strengths and Limitations 

The study presents some limitations. First of all, it includes a limited number of respondents. However, the population included more than 400 staff members from four different NHs, and the low RR itself might be seen as significative result. In addition, a strength of the study is that not only HCWs but also all other professions working in the NH were involved.

Secondly, it was not possible to directly compare the population of unvaccinated versus vaccinated participants due to the low number of respondents from the latter group. However, analysing the determinants of participants’ hesitancy allowed us to explore several critical aspects negatively affecting positive attitudes towards vaccination. Moreover, the use of the 3Cs model gave us the opportunity to interpret the results according to a well-recognised framework and helped us to identify challenging areas that need to be improved in order to overcome hesitancy.

Finally, the convenience dimension showed low reliability properties. Indeed, this latent factor included items exploring a few aspects affecting in different ways the access to flu shots. However, confirmatory factor analysis demonstrated the satisfactory robustness of the model. Moreover, considering that our research aims were mainly explorative, we assessed only the content validity and internal consistency to determine to which extent the questionnaire items represented all facets (i.e., confidence, complacency and convenience) of the given construct (i.e., the 3C SAGE theoretical model). If the questionnaire is used for other research purposes (e.g., exploring healthcare providers’ attitudes over time or after an intervention), some additional analyses will be required in order to assess, for instance, its stability over time (i.e., test–retest) or generalizability (e.g., external validation with a golden standard).

## 5. Conclusions

Most flu vaccination campaigns focus either on vaccine efficacy or on safety. However, according to our results, the strongest determinant was the perception of risks related to the disease. An effective program to promote vaccination uptake by NH personnel should therefore seek to reduce complacency. For example, sensitizing staff towards the potentially severe consequences of influenza even for elderly people who regularly get vaccinations (due to immune system senescence) may help to raise NH staff’s commitment to this essential prevention strategy. Exploring staff attitudes towards flu vaccination was found to be a useful tool to identify educational and informational needs and guide managers in the development of adequate, tailored interventions able to increase the extremely low vaccination coverage.

## Figures and Tables

**Table 1:** Responder description and response rate (RR) per staff position (*n* = 166).

Staff Position	Respondents	RR *
Staff managers/Leadership—Administrator, Medical Director, Director of Nursing	5 (3.0%)	33.3% (5/15)
Direct care staff—Physicians ^1^, Healthcare Assistants, Healthcare Technicians, physical therapists	88 (53.0%)	34.7% (88/254)
Nurses	31 (18.7%)	64.6% (32/48)
Other healthcare providers—Occupational/Speech/Respiratory therapists, dieticians/nutritionists, animators, Social Worker, Psychologist	18 (10.8%)	78.3% (18/23)
Administrative staff	9 (5.4%)	42.9% (9/21)
Support staff—Food Service/Dietary, Housekeeping, Laundry Service, Maintenance	8 (4.8%)	10.5% (8/76)
Missing	7 (4.2%)	

^1^ Physicians were included in the direct care staff group due to their low number in the whole sample (*n* = 5); * RR calculated as the number of respondents over the total number of recruited per each staff position.

**Table 2:** Frequency distribution (%) of items representing the 3Cs of the model.

Latent Factors	Items	*n*	Score (%) ^1^				
			1	2	3	4	5
**Confidence**	Being vaccinated protects against flu (Item 1)	155	11.6	21.9	43.2	17.4	5.8
	The flu vaccine is effective (Item 2)	155	9.7	15.5	50.3	22.6	1.9
	I don’t think flu vaccination is useful (Item 7)	153	12.4	24.8	37.9	18.3	6.5
	I’m concerned about the adverse events of the influenza vaccine (Item 8)	153	9.2	24.8	35.3	24.2	6.5
**Complacency**	It is important to protect my family against the flu by vaccinating myself (Item 3)	153	10.5	17.6	44.4	23.5	3.9
	It is important to protect residents against the flu by vaccinating myself (Item 4)	151	9.9	15.2	44.4	25.8	4.6
	The risk of getting the flu is very high in the nursing home setting (Item 5)	153	5.2	22.9	30.7	36.6	4.6
	The flu disease is a potentially very severe/dangerous condition (Item 6)	153	4.6	13.7	39.9	37.2	4.6
**Convenience**	I have been adequately informed by a physician about the benefits of the influenza vaccination (Item 9)	153	7.8	21.6	26.8	39.9	3.9
	I do not regularly get the influenza vaccination due to forgetfulness or lack of time (Item 10)	148	31.8	36.5	22.3	5.4	4.0
	It would be useful to make the influenza vaccination mandatory for health professionals working in NHs (Item 11)	153	35.9	31.4	21.6	7.8	3.3
	In the NH where I work, it is easy to get the influenza vaccination (Item 12)	154	2.6	1.9	18.8	48.7	27.9

^1^ 1 = Strongly disagree; 2 = disagree; 3 = neither agree nor disagree; 4 = agree; 5 = strongly agree. Item 7, Item 8 and Item 10 are negatively worded and scores need to be reversed (e.g., proportion of answers 1 and 2 at the Likert scale correspond to the positive scores, while proportion of answers 4 and 5 to negative scores). NH: nursing home.

**Table 3:** Determinants of the attitudes towards vaccination. CI: confidence interval.

Predictors	Simple Regressions			Multiple Regression		
	Beta	*p*-Value	95% CI	Beta	*p*-Value	95% CI Beta
Being advised by a physician (Q2)	−0.16	0.36	−0.50; −0.18			
Working with residents (Q3)	−0.38	0.14	−0.87; 0.12			
Complacency	0.86	<0.01	0.68; 1.04	0.60	<0.01	0.36; 0.84
Confidence	0.67	<0.01	0.49; 0.85	0.25	0.02	0.03; 0.46
Convenience	0.76	<0.01	0.45; 1.06	0.38	<0.01	0.11; 0.65

## Appendix A

                    **SURVEY.**

                **Flu vaccination among the NHs staff.**

**Q1. How many times did you get vaccinated for influenza in the last 3 years?**

  ☐ 0  ☐ 1  ☐2  ☐3

**Q2. Have you ever been advised by a physician to get the flu vaccination?**

  ☐ Yes ☐ No

**If yes, what was the main reason you were suggested to get the flu vaccine?**

**(You are allowed to select more than 1 answer)**

☐ Chronic disease  ☐ Work risk category  ☐ Other______________________________

**Q3. What is your job in this nursing home? Check ONE box that best applies to your job. If more than one category applies, check the highest-level job.**

☐ Staff managers/Leadership**-***Administrator, Medical Director, Director of Nursing*

☐ Physicians

☐ Direct care staff-*Healthcare Assistants, Healthcare Technicians, physical therapists*

☐ Nurses

☐ Other healthcare provider-*Occupational/Speech/Respiratory therapists, dieticians/nutritionists, animators, Social Worker, Psychologist*

☐ Administrative staff

☐ Support staff-Food Service/Dietary, Housekeeping, Laundry Service, Maintenance

☐ Other (Please, specify ___________________________________)

**Q4. Please indicate how much you agree or disagree with the with the following statements? Please select for each item a score on the scale, ranging from 1 (“strong disagreement”) to 5 (“Strong agreement”).**

	**Strong Disagreement** **▼**	**Disagreement** **▼**	**Neither in Agreement, Nor in Disagreement** **▼**	**Agreement** **▼**	**Strong Agreement** **▼**
1. Being vaccinated protects against influenza	☐1	☐2	☐3	☐4	☐5
2. The influenza vaccine is effective	☐1	☐2	☐3	☐4	☐5
3. It is important to protect my family against the flu by vaccinating myself	☐1	☐2	☐3	☐4	☐5
4. It is important to protect residents against the flu by vaccinating myself	☐1	☐2	☐3	☐4	☐5
5. The risk of getting influenza in NHs is very high.	☐1	☐2	☐3	☐4	☐5
6. Influenza is a potentially very dangerous/severe condition	☐1	☐2	☐3	☐4	☐5
7. I do not think that the influenza vaccination is useful	☐1	☐2	☐3	☐4	☐5
8. I am concerned about the adverse events of the influenza vaccine	☐1	☐2	☐3	☐4	☐5
9. I have been adequately informed by a doctor about the benefits of the influenza vaccination	☐1	☐2	☐3	☐4	☐5
10. I do not regularly get the influenza vaccination due to forgetfulness or lack of time	☐1	☐2	☐3	☐4	☐5
11. It would be useful to make the influenza vaccination mandatory for health professionals working in the NH	☐1	☐2	☐3	☐4	☐5
12. In the NH where I work, it is easy to get the influenza vaccination.	☐1	☐2	☐3	☐4	☐5
13. Staff working in the NH should get the flu vaccination each year	☐1	☐2	☐3	☐4	☐5

## Appendix B

**Table A1:** Score distribution of the never-vaccinated group (i.e., 0 at Q1) compared to the vaccinated at least once group (i.e., 1–3 for Q1).

Items		Never Vaccinated						Vaccinated at Least Once				
	*n*	1	2	3	4	5	*n*	1	2	3	4	5
Item 1. Being vaccinated protects against influenza	129	13.2	24.8	45.0	11.6	5.4	16	0.0	0.0	37.5	43.8	18.8
Item 2. The influenza vaccine is effective	129	10.9	17.8	51.2	18.6	1.6	16	0.0	0.0	56.3	37.5	6.3
Item 3. It is important to protect my family against the flu by vaccinating myself	128	11.7	19.5	46.1	19.5	3.1	16	0.0	0.0	31.3	56.3	12.5
Item 4. It is important to protect residents against the flu by vaccinating myself	127	11.0	16.5	46.5	22.0	3.9	16	0.0	0.0	25.0	62.5	12.5
Item 5. The risk of getting influenza in NHs is very high	128	5.5	22.7	32.8	35.2	3.9	16	0.0	25.0	12.5	50.0	12.5
Item 6. Influenza is a potentially very dangerous/severe condition	128	3.9	14.8	37.5	39.1	4.7	16	0.0	0.0	56.3	37.5	6.3
Item 7. I do not think that influenza vaccination is useful	128	10.2	21.9	41.4	18.8	7.8	16	25.0	56.3	18.8	0.0	0.0
Item 8. I am concerned about the adverse events of the influenza vaccine	129	8.5	23.3	37.2	24.0	7.0	15	20.0	40.0	26.7	13.3	0.0
Item 9. I have been adequately informed by a doctor about the benefits of the influenza vaccination	129	8.5	22.5	27.1	38.0	3.9	15	6.7	6.7	20.0	60.0	6.7
Item 10. I do not regularly get the influenza vaccination due to forgetfulness or lack of time	124	33.1	37.1	20.2	4.8	4.8	15	33.3	33.3	26.7	6.7	0.0
Item 11. It would be useful to make the influenza vaccination mandatory for health professionals working in the NH	128	20.3	34.4	36.7	5.5	3.1	16	6.3	0.0	50.0	31.3	12.5
Item 12. In the NH where I work, it is easy to get the influenza vaccination.	129	40.3	34.9	18.6	3.1	3.1	16	12.5	12.5	37.5	31.3	6.3
Item 13. Staff working in the NH should uptake flu vaccination each year	129	3.1	1.6	17.8	48.8	28.7	16	0.0	0.0	18.8	43.8	37.5
